# ST-Elevation Myocardial Infarction in Coronary Ectasia: A Case Report

**DOI:** 10.3390/diseases6040104

**Published:** 2018-11-16

**Authors:** Hye Ji (Sally) Choi, Christina Luong, Anthony Fung, Teresa S. M. Tsang

**Affiliations:** Division of Cardiology and Cardiovascular Surgery, Vancouver General Hospital, University of British Columbia, Vancouver, BC V6T 1Z4, Canada; sallyhyejichoi@gmail.com (H.J.C.); cluong@ualberta.ca (C.L.); a.fung@ubc.ca (A.F.)

**Keywords:** coronary artery ectasia, STEMI, atherosclerosis, case report

## Abstract

Coronary artery ectasia (CAE) is localized or diffuse dilatation of the coronary artery lumen exceeding the diameter of adjacent healthy reference segments by 1.5 times. It is a rare phenomenon and incidence ranges from 1 to 5% in patients undergoing angiography. We report a case of a 58-year-old man with atherosclerotic CAE who experienced ST-elevation myocardial infarction (STEMI) despite prophylactic antiplatelet therapy. He was successfully treated with IV eptifibatide and aspiration thrombectomy. We reviewed the literature of CAE presentation, etiology and treatment and discussed the most appropriate antithrombotic therapy to prevent STEMIs in patients with CAE. While the current literature appears to favour prophylactic antiplatelet and anticoagulant in these patients, more studies are needed to determine the optimal form and duration of antithrombotic therapy. Currently, there is no gold standard treatment for CAE and further prospective and randomized-controlled studies are needed to guide recommendations.

## 1. Case Report

**Figure 1:**
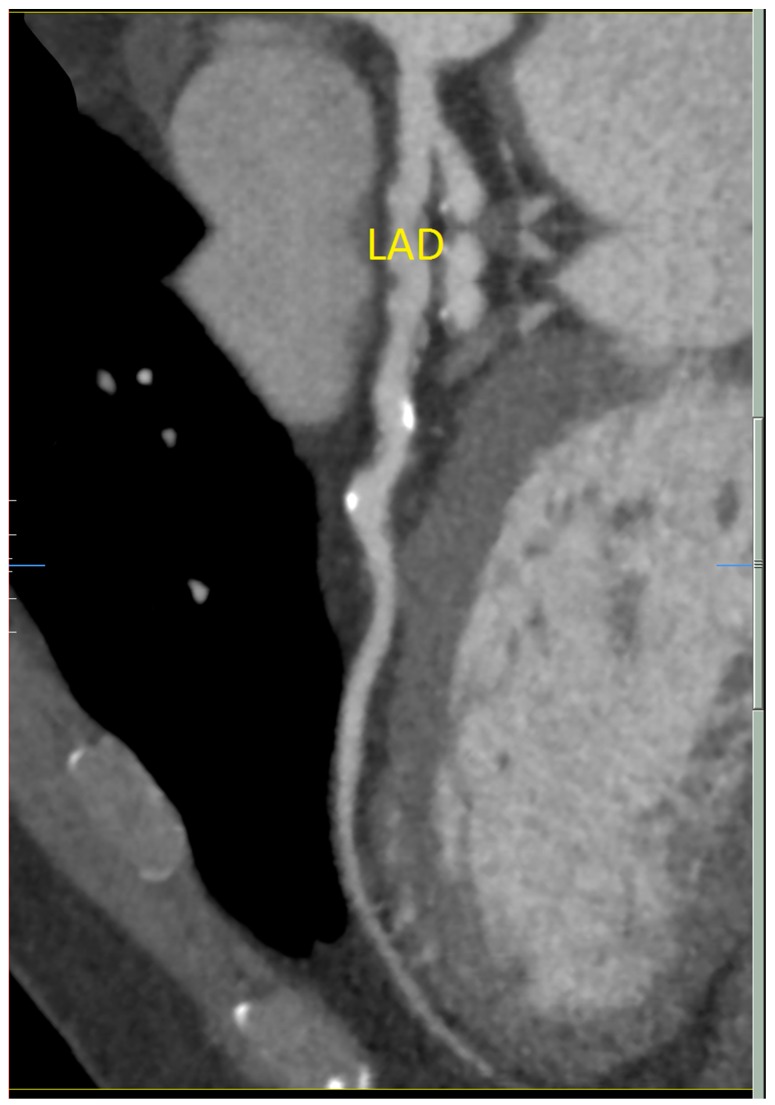
Cardiac computed tomography showing diffuse ectasia of the left anterior descending (LAD) artery.

A 58-year-old man presented to medical attention with atypical chest tightness with exertion. He has a history of hypertension, hyperlipidemia, and orthostatic dizziness/pre-syncope. He has no history of diabetes and is a non-smoker. He underwent investigations including an electrocardiogram (ECG) and an exercise stress test, which were both normal. Echocardiogram was unremarkable as well, revealing mildly concentrically increased left ventricular wall thickness and mild biatrial enlargement. Cardiac computed tomography demonstrated diffuse non-obstructive coronary artery disease (CAD). The right coronary artery (RCA) was shown to have a cylindrical aneurysmal dilatation over 38 mm with associated plaque and the proximal left anterior descending (LAD) artery had an ulcerated noncalcified plaque (25–49%) (Figure 1). He has no specific family history for premature CAD, arrhythmia or sudden cardiac death.

As a result of these findings, he underwent coronary angiographic assessment to clarify his anatomy. At the cardiac catheterization laboratory, a right radial artery access was obtained and subsequent left heart catheterization and left ventriculogram revealed left-ventricular end-diastolic pressure (LVEDP) of 15 mmHg and normal left-ventricular ejection fraction (LVEF) with normal wall motion. Coronary angiography revealed a right-dominant system with ectasia involving the proximal RCA, LAD artery and left circumflex (LCx) artery with no obstructive lesions. The ectasia was thought to be most likely related to atherosclerosis. The patient was started on atorvastatin (10 mg/day), candesartan (4 mg/day), and aspirin (81 mg/day). No anticoagulant therapy was recommended. 

**Figure 2:**
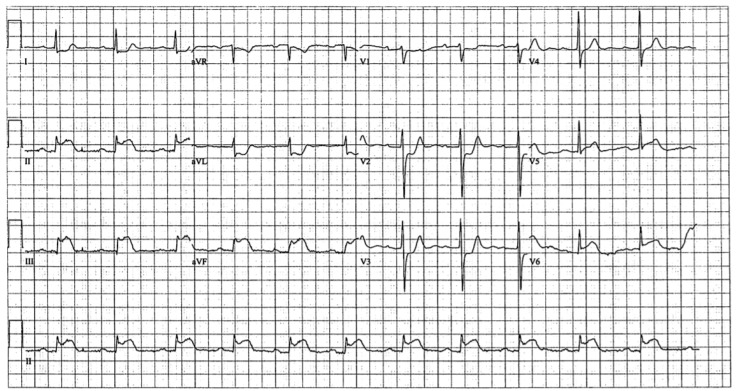
Electrocardiogram showing inferior ST-elevation myocardial infarction.

**Figure 3:**
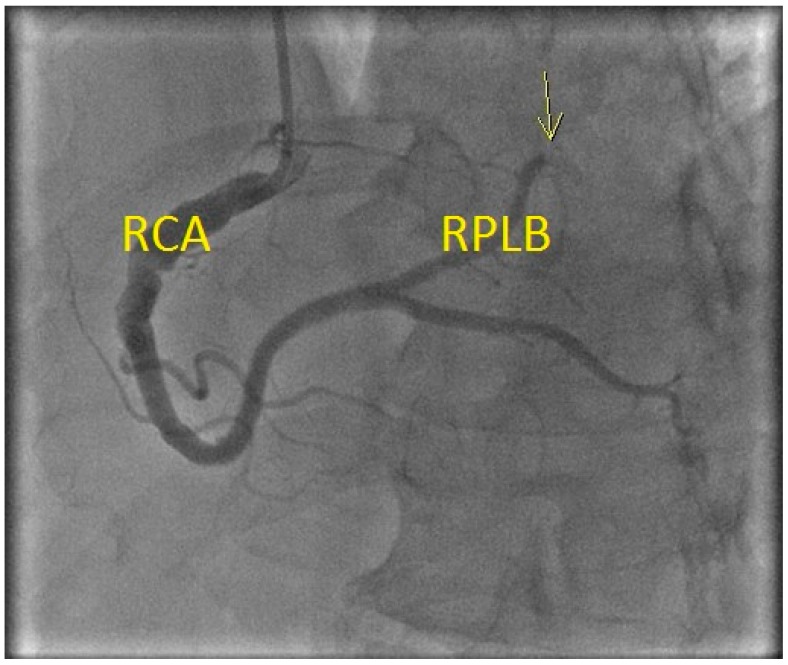
Coronary angiogram showing thrombus at the proximal right coronary artery (RCA) with occlusion (yellow arrow) of the distal right posterolateral branch (RPLB).

Four months later, the patient experienced chest pain while playing squash. Vitals were unremarkable and only the blood pressure was mildly elevated at 141/103. ECG done in the field showed inferior ST-elevation myocardial infarction (STEMI) (Figure 2). The cardiac catheterization laboratory was activated. The left heart catheterization and ventriculogram revealed normal LV ejection fraction with mild inferior hypokinesia. Coronary angiography revealed thrombus at the proximal RCA with occlusion of the distal right posterolateral branch and distal right posterior descending artery due to embolism, without associated stenotic lesions (Figure 3). He was treated with intravenous (IV) eptifibatide (22.5 mL bolus × 2, 15 mL/h infusion) and heparin (2000 units), followed by aspiration thrombectomy. Stenting was deferred due to the ectatic nature of his vessels. Thrombectomy was performed on the lesion at the 1st right poster olateral segment. Using a 6FR Runway FR4 guiding catheter, BMW Balance Middle Weight wire was used to cross the lesion. Balloon angioplasty was performed using a Sprinter Legend RX 1.5 × 20 balloon, with 1 inflation at a maximum of 10 atm pressure. Two attempts of mechanical thrombectomy were performed, with a maximum duration of 56 s and a volume of 30 mL. Visible thrombus was retrieved but the distal branches remained occluded. Echocardiogram confirmed hypokinesis of the inferior wall. The patient was treated with IV eptifibatide for 24 h and IV heparin for 48 h before being discharged in a stable condition. He was discharged on Clopidogrel (75 mg/day) and Rivaroxaban (15 mg/day) in addition to his preadmission risk modifying medications. The patient was adherent to the medications and there were no reported adverse events. A follow-up cardiac CT (2 months later) revealed no residual coronary artery thrombus, high-grade stenosis or occlusion and the latest treadmill stress test (11 months later) was negative for ischemia as well. 

## 2. Discussion

Coronary artery ectasia (CAE) is an uncommon condition with localized or diffuse dilatation of the coronary artery lumen exceeding the diameter of adjacent healthy reference segments by 1.5 times [1,2]. Patients can be asymptomatic or present with atypical chest pain, stable angina or acute coronary syndromes [2]. Coronary angiography is used to diagnose CAE [3], and the incidence ranges from 1 to 5% in patients undergoing angiography [1]. Hypertension and dyslipidemia are some of the risk factors while diabetes mellitus has been inversely associated with the incidence of CAE [4].

The full etiology of CAE is not elucidated, but CAE is often found in association with conditions such as Kawasaki disease or familial hypercholesterolemia [4]. The most common association is CAD and about 85% of CAE patients also have coronary atherosclerosis [2]. In fact, retrospective and prospective studies have established an appreciable incidence of myocardial infarctions in CAE patients and hence, aspirin is routinely advised to all CAE patients [1,2,4]. Atherosclerotic CAE has similar morbidity and mortality rates and risk factors as CAD alone and also has common risk factors [5]. Hence, atherosclerotic CAE management is modelled after CAD management, including regular aspirin therapy [1] and aggressive risk factor modifications such as cholesterol level control [5]. Medications such as statins, angiotensin-converting enzyme inhibitors, angiotensin II receptor blockers, and dihydropyridine calcium channel blocker have been shown to be useful in the management of CAE [5]. However, most treatment recommendation are based on expert opinion and no official guidelines exist [5].

Our patient with atherosclerotic CAE experienced a thrombus-induced STEMI despite prophylactic antiplatelet treatment. Literature is unclear on whether additional prophylactic anticoagulant therapy would have prevented this outcome. Flow disturbances have been observed in ectatic regions of coronary arteries, and hence, some authors have proposed chronic anticoagulation as a solution [1]. However, there has been no prospective data to support this therapy and treatment must be individualized until further evidence is available [1]. Some suggest that chronic anticoagulation for primary prevention of MI should only be reserved for patients with severe coronary artery dilation (≥2-fold or ≥8 mm), since this group experiences higher morbidity and mortality rates [5]. While the current wisdom appeared to favor lifetime anticoagulant in these patients, the evidence is still sparse and more studies are needed to determine the optimal form and duration of antithrombotic therapy. The value of lifetime anticoagulant therapy for secondary prevention is less ambiguous and patients who experienced a thrombosis-induced MI should be initiated on this therapy after assessing the patient for bleeding risk [5].

Our STEMI patient was successfully treated with IV eptifibatide and aspiration thrombectomy. Other strategies for treating thrombotic occlusions include heparin infusions and fibrinolysis [1]. If medical treatment fails, percutaneous or surgical coronary re-vascularization should be considered [1]. It is important to note that percutaneous treatments such as stent implantation or aspiration thrombectomy can cause distal thromboembolism [5]. Although CAE is a strong independent predictor of no-reflow phenomenon after PCI for STEMI and alternative treatment strategies may be needed, revascularization rates and survival are comparable to those in patients without CAE [6,7]. Surgical techniques include resection, ligation, and bypass grafting, and many have been successful, although it is unclear which, if any, is superior [5]. In our patient, balloon angioplasty was performed without stenting due to the diffuse ectatic nature of the vessels. However, stenting can be a successful treatment method in focal ectasia and polytetrafluoroethylene-covered or self-expanding stents are some options for sealing off aneurysmal segments [3]. In conclusion, as of 2018, there is no gold standard treatment for CAE and further prospective and randomized-controlled studies are needed to guide recommendations. 

Consent has been obtained from the patient. 

## 3. Key Points

1. Coronary artery ectasia (CAE) is an uncommon condition with localized or diffuse dilatation of the coronary artery lumen exceeding the diameter of adjacent healthy reference segments by 1.5 times.
2. Atherosclerotic CAE has similar morbidity and mortality rates and risk factors as CAD and therefore, atherosclerotic CAE management is modelled after CAD management, including regular aspirin therapy and aggressive risk factor modifications.
3. While the current wisdom favors lifetime anticoagulant in CAE patients, the evidence is still sparse and more studies are needed to determine the optimal form and duration of antithrombotic therapy.
4. Stenting for treatment of thrombotic occlusions in patients with diffuse ectasia can pose a challenge and alternative treatment methods should be explored.
